# Current progress in CAR-based therapy for kidney disease

**DOI:** 10.3389/fimmu.2024.1408718

**Published:** 2024-08-20

**Authors:** Dan Zhang, Dong Sun

**Affiliations:** ^1^ Department of Nephrology, Affiliated Hospital of Xuzhou Medical University, Xuzhou, China; ^2^ Department of Internal Medicine and Diagnostics, Xuzhou Medical University, Xuzhou, China; ^3^ Clinical Research Center For Kidney Disease, Xuzhou Medical University, Xuzhou, China

**Keywords:** chimeric antigen receptor T cell therapy, kidney disease, cell immunotherapy, autoimmune diseases, T lymphocytes

## Abstract

Despite significant breakthroughs in the understanding of immunological and pathophysiological features for immune-mediated kidney diseases, a proportion of patients exhibit poor responses to current therapies or have been categorized as refractory renal disease. Engineered T cells have emerged as a focal point of interest as a potential treatment strategy for kidney diseases. By genetically modifying T cells and arming them with chimeric antigen receptors (CARs), effectively targeting autoreactive immune cells, such as B cells or antibody-secreting plasma cells, has become feasible. The emergence of CAR T-cell therapy has shown promising potential in directing effector and regulatory T cells (Tregs) to the site of autoimmunity, paving the way for effective migration, proliferation, and execution of suppressive functions. Genetically modified T-cells equipped with artificial receptors have become a novel approach for alleviating autoimmune manifestations and reducing autoinflammatory events in the context of kidney diseases. Here, we review the latest developments in basic, translational, and clinical studies of CAR-based therapies for immune-mediated kidney diseases, highlighting their potential as promising avenues for therapeutic intervention.

## 1 Introduction

The conventional classification of kidney diseases has historically relied on the histological patterns of kidney injury. However, the past decade has experienced remarkable breakthroughs that have reshaped our comprehension of the pathogenesis, diagnosis, and treatment of glomerular diseases. Notably, the identification of antibodies against phospholipase A2 receptor (anti-PLA2R antibodies) has revealed membranous nephropathy (MN) as an autoimmune disease (1). Moreover, it has become increasingly evident that complement activation plays a pivotal role in various glomerular diseases, with the alternative pathway implicated in IgA nephropathy (IgAN) and C3 glomerulopathy, the lectin pathway in IgAN and lupus nephritis (LN), and the terminal pathway in complement-mediated thrombotic microangiopathy (TMA) (2). These significant discoveries emphasize the increasing complexity of understanding glomerular disease etiology and offer substantial prospects for targeted therapeutic interventions.

The interdependence between the kidneys and immune system is evident as the kidneys contribute to maintaining immunological homeostasis through hormone production and the presence of resident immune cells. This interplay makes the kidneys susceptible to direct or indirect immune system attacks. The intricate nature of immunity contributes to the diverse clinical presentations and histopathological manifestations observed in glomerular diseases. Currently, most glomerular diseases are managed with a combination of high-dose corticosteroids and nonspecific immunosuppressive agents such as cyclophosphamide, azathioprine, cyclosporine, or mycophenolate-mofetil to induce remission (3). However, these treatments are not tailored to specific types of glomerulonephritis, and many patients experience adverse effects. In contrast, the use of new biologic agents offers significant advantages by targeting specific molecules and providing more targeted therapy to patients (4). Nonetheless, a subset of patients exhibit a poor response or are diagnosed with refractory renal disease (5). Thus, despite substantial advancements, novel therapeutic strategies for immune kidney conditions are urgently needed.

Cell-based immunotherapies, which span from the field of blood malignancies to autoimmune diseases, have captured increasing attention from researchers. CAR T-cell therapy is a recent and rapidly developing treatment modality involving the genetic engineering of a patient’s T cells while using a retrovirus or lentivirus to introduce a CAR fusion protein (6) (7),. These CAR T cells can then target and destroy cells that express a specific antigen, leading to strong T-cell activation and potent antitumor responses. This therapy has shown remarkable efficacy in treating hematological malignancies such as lymphoma, leukemia, and multiple myeloma, as well as some solid tumors. Some patients have achieved long-term remission and, in some cases, even a potential cure (8–11).

Although CAR T cells were originally developed for cancer treatment, they hold promising potential as novel therapeutic approaches for immune-mediated kidney diseases. These cells possess the inherent ability to specifically target and eliminate immune cells that have become pathologically activated, or restore immune tolerance in organs affected by dysregulated immunity (12–14). For example, the development of chimeric autoantigen receptor (CAAR) T cells capable of expressing relevant autoantigens represents a potential strategy to attract autoreactive B cells. These CAAR T cells can bind to autoreactive B cells through specific interactions between the autoantigen and the target B-cell receptor. This approach represents an advancement over the current nonselective B cell depletion therapy using rituximab. Alternatively, CAR T cells could serve as Tregs to restore tolerance in immune glomerular diseases. Nevertheless, this remains an emerging area of research, and further studies are warranted to assess the safety and efficacy of CAR T cells in the context of kidney diseases. Thus, in this review, we aim to summarize and evaluate the current state of research on the use of CAR T-cell therapy in managing kidney diseases, focusing on exploring the potential benefits and limitations of CAR T-cell therapy.

## 2 Pathogenesis of immune-mediated kidney diseases

**Figure 1:**
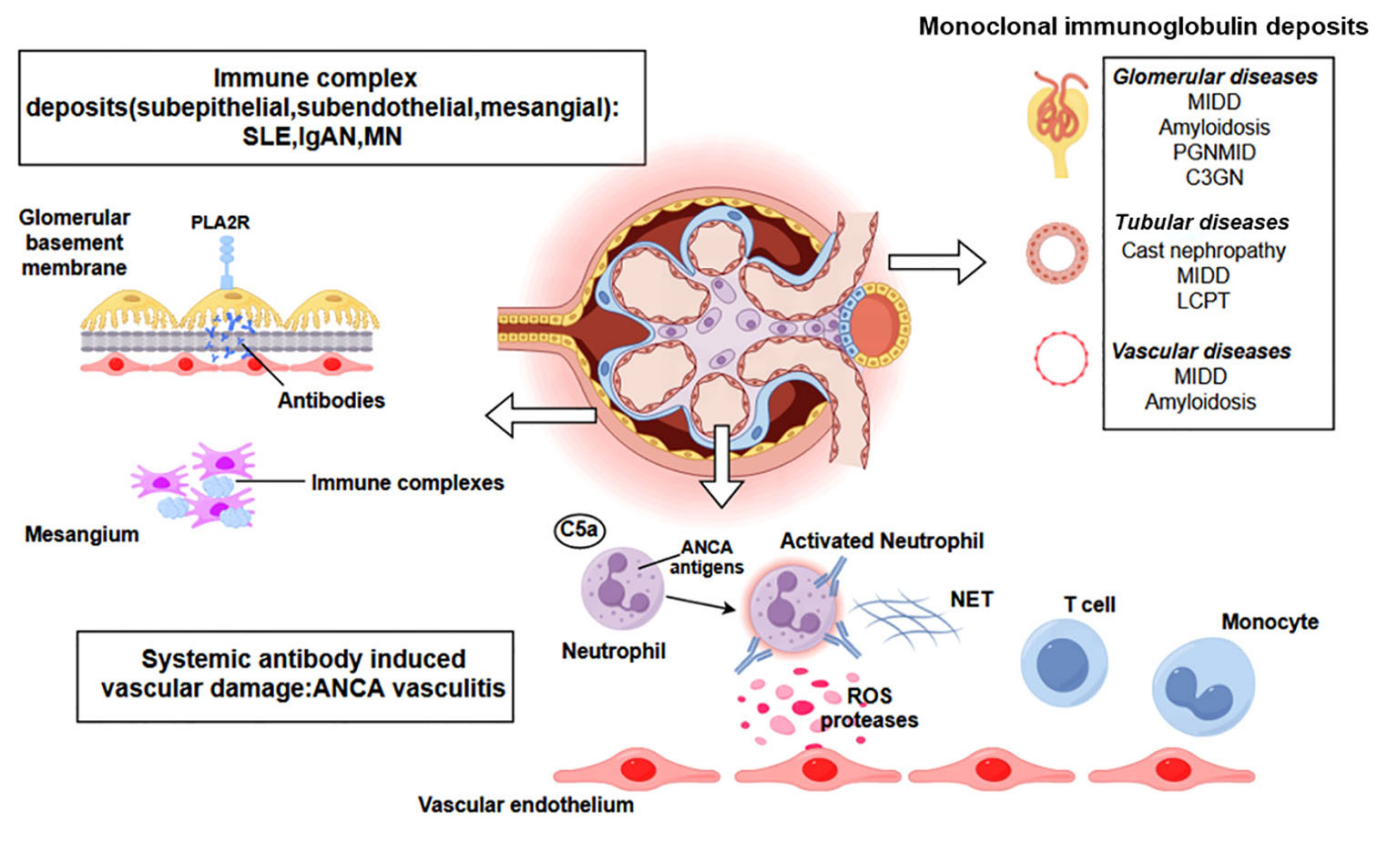
A representation of various immune-mediated kidney diseases including systemic antibody-induced vascular damage (ANCA-associated vasculitis), immune complex deposits (SLE, IgAN, MN), monoclonal immunoglobulin deposits. Additional abbreviations: ANCA, antineutrophil cytoplasmic antibody; SLE, systemic lupus erythematosus; IgAN, IgA nephropathy; MN, Membranous Nephropathy; PLA2R, M-type phospholipase A2 receptor1; C3GN, C3 glomerulonephritis; LCPT, light chain proximal tubulopathy; MIDD, monoclonal immunoglobulin deposition disease; PGNMID, proliferative glomerulonephritis with monoclonal immunoglobulin deposits; ROS, reactive oxygen species; NET, neutrophil extracellular trap. Created with figdraw.com.

Kidney diseases linked to immune homeostasis disruption can be categorized as either direct or indirect immune-mediated renal injury. Anders HJ et al. (15) proposed grouping immune-mediated disorders into five categories according to their immunopathogenesis: infection-related GN, autoimmune GN, alloimmune GN, autoinflammatory GN and monoclonal gammopathy-related GN. This categorization can inform the appropriate treatment. As shown in **Figure 1**, several major mechanisms are involved in immune-mediated kidney injury. In direct immune-mediated kidney disease, the immune system targets specific kidney antigens, whereas in indirect cases, the kidneys are bystander victims of systemic immune dysregulation. Cellular injury in glomerular disease is caused by elements from both the innate and adaptive immune systems (16). Secondary events triggered by the immune response, such as complement activation, inflammatory cell infiltration, and the activation of intrinsic renal cells with the release of inflammatory mediators further exacerbate the kidneys, leading to glomerular inflammation or sclerosis and causing various clinical symptoms.

### 2.1 Cell-mediated immunity

Evidence suggests that mononuclear cells, especially lymphocytes and macrophages, play a primary role in the development of conditions such as minimal change disease (MCD), focal segmental glomerulosclerosis (FSGS), and crescentic glomerulonephritis, even in the absence of antibody deposition (17). T cells play a pivotal role in autoimmune diseases, employing various effector mechanisms to mediate renal damage and repair, including local cytokine production, which relies on their localized presence at the site of inflammation. Initial CD4+ T cells recognize major histocompatibility complex class II (MHC-II), which is presented by antigen-presenting cells (APCs), initiating cellular immunity and expressing high-affinity receptors for interleukin (IL)-2. Simultaneously, the costimulatory molecule B7 (CD80/86) on APCs interacts with the CD28 receptor on T cells, activating CD4+ T cells. Activated CD4+ T cells proliferate under the influence of IL-2 and differentiate into subsets of helper T cells (Th cells), e.g., TH1, TH2, TH17, and T follicular helper (TFH) cells, and thus far, less well-characterized subtypes, such as IL-9-producing TH9 cells and TH22 cells, as well as Treg cells, produce diverse biological effects (18). In particular, TH1 and TH17 cells have been implicated in the pathogenesis of immune-mediated glomerular diseases, whereas Treg cells regulate excessive immune responses (19).

### 2.2 Humoral immunity

The humoral immune response is the main mechanism of injury in immune-mediated glomerulonephritis. Evidence suggests that autoantibodies and autoreactive B cells are the underlying or contributing factors in the pathogenesis of immune-mediated glomerulonephritis (20, 21). Some self-antigens that trigger immune-mediated glomerulonephritis have been identified (22). B cells play a critical role in humoral immunity through antibody production, complement system activation, and their interaction with T cells via antigen presentation, costimulatory signals, and cytokine-mediated modulation (20). Mature B cells differentiate into plasma cells, plasma blasts, and memory B cells when they recognize (auto)antigens and receive appropriate T cells help. Both plasma blasts and plasma cells are responsible for producing and secreting antibodies. The transition between these stages involves the differential expression of surface markers and transcription factors. The roles of B cells in humoral immunity include: (1) producing antibodies that bind to antigens and induce complement system activation or antibody-dependent cellular cytotoxicity (ADCC), (2) serving as antigen-presenting cells (APCs) to induce T-cell activation and memory cell differentiation, and (3) secreting proinflammatory cytokines such as tumor necrosis factor (TNF), IL-6, and granulocyte-macrophage colony-stimulating factor (GM-CSF), which activate myeloid cells and induce T-cell differentiation (20).

An essential pathway for B-cell differentiation, survival, and activation involves two ligands from the TNF superfamily, BlyS (also known as Baff) and April, and their corresponding B-cell receptors BCMA, TACI, and BAFF-R (specifically for BlyS) (23). These three receptors collectively augment NF-κB, thereby promoting the activation and differentiation of B cells. Biologics targeting these B-cell activation molecules have been used to treat various autoimmune diseases (24).

### 2.3 Complement activation

The complement cascade and complement regulatory system also play crucial roles in the occurrence and development of immune-mediated glomerulonephritis (25). The primary pathological driver of atypical hemolytic uremic syndrome and C3 glomerulonephritis, conditions that arise from uncontrolled complement activation, is attributed to genetic alterations within the complement regulatory proteins or the presence of autoimmune responses targeting complement system constituents (2). Following a triggering event, such as infection, pregnancy, medication use, or transplantation, and superimposed on a genetic predisposition, the alternative complement pathway is dysregulated and overactivated. This excessive activation results in the formation of membrane attack complexes, causing endothelial cell injury (26).

Kidney diseases involving complement include IgAN, LN, and MN, as well as ANCA-associated vasculitis and focal segmental glomerulosclerosis (2). In LN, the complement system plays a dual role. On the one hand, genetic alterations within the initial components of the classical complement cascade are identified as susceptibility factors. On the other hand, the activation of complement by immune complexes, leading to tissue destruction, is a defining characteristic of SLE (27). Immune complexes are known to initiate the classical complement pathway, whereas apoptotic materials can trigger the lectin pathway. Studies have indicated that activation of the alternative complement pathway is instrumental in the etiology of LN and provides a more precise reflection of disease activity (27, 28). In MN, antibodies against the M-type phospholipase A2 receptor (PLA2R), which exhibits glycosylation abnormalities, are capable of engaging in the lectin pathway, whereas, in a minority of patients, anti-complement factor H (CFH) antibodies activate the alternative pathway (29, 30). Recent studies have suggested the substantial involvement of C3a and its receptor in disease pathogenesis, suggesting that C3a is a promising target for new therapeutic strategies (31). The activation of the alternative complement pathway plays a main role in the pathogenesis of ANCA-associated vasculitis, highlighting the critical importance of C5a and its receptors (32). Recent studies have shown that several complement-targeted drugs have been approved for the treatment of kidney disease, and additional anti-complement agents are being investigated in clinical trials (2).

### 2.4 Neutrophils

Numerous studies implicate neutrophils as pivotal contributors to the pathogenesis of immune-mediated kidney diseases, exerting their influence through the formation of neutrophil extracellular traps (NETs), the secretion of proinflammatory cytokines, and the facilitation of tissue destruction (33). Once released into the extracellular space, components of NETs can act as autoantigens, causing a break in self-tolerance. This cascade can ultimately precipitate the emergence of autoimmune reactions in susceptible individuals. Additionally, dysregulation of NET formation and degradation could extend the immune system’s exposure to these altered autoantigens, thereby increasing the degree of tissue damage induced by NETs. Neutrophil degrading enzymes can directly trigger endothelial cell apoptosis and degrade the basement membrane; whereas tubulointerstitial injury reduces glomerular blood flow, creating a NETotic environment (34–36). Epithelial tubular cells release histones in response to hypoxia and kidney injury, which subsequently stimulate neutrophils to release more NETs. This initiates a proinflammatory cascade that perpetuates endothelial cell damage. Indirectly, NETs exacerbate vascular injury by activating the alternative complement pathway and lead to kidney fibrosis by facilitating inflammation and the macrophage-to-myofibroblast transition (37).

Neutrophil priming is considered to be the key step in NET formation in AAV. Priming is a process in which the neutrophil response is enhanced by an activating stimulus. After neutrophil activation, MPO and PR3 are translocated to the cell surface. ANCAs crossbind these antigens with neutrophil FcgRIIa and induce uncontrolled ROS and lytic enzyme bursts. Following the burst, MPO and NE migrate to the nucleus, thereby initiating and perpetuating the NETosis process through an NADPH-oxidase-dependent pathway, via the lytic NETosis mechanism (38).

## 3 CAR T cell technology

CAR T cells, engineered T cells designed to target a specific antigen (39), consist of a structure that is a fusion of the antibody structure and the T-cell receptor (TCR). Zelig Eshhar et al. first proposed the use of a CAR to guide specific T cells almost 30 years ago (40, 41). The use of CAR T- cell therapy has demonstrated significant effectiveness in treating leukemia (42, 43). Recent advancements indicate that CAR T-cell therapy holds tremendous promise for the treatment of various types of autoimmune kidney diseases, which are limited by abnormal attack by the immune system, which targets renal tissues.

The typical synthetic single-chain antigen receptor of the CAR product comprises three structural domains, namely an ectodomain, a transmembrane domain, and an endodomain (44). The specific recognition of antigens by the receptor is conferred by the antigen-binding domain, which is primarily composed of the variable heavy (VH) and light (VL) chains of antibodies, linked by a flexible linker to form a single-chain variable fragment (scFv) (44). Moreover, alternative molecules, including nanobodies, cytokine-binding proteins, and other high-affinity domains for target antigens, have also been employed as antigen-binding domains for the extracellular segment of CARs (45). CARs have advanced through four generations. The initial generation of CARs combine scFv domains with the CD4 extracellular domain and the CD3ζ intracellular domain, resulting in insufficient stability and clinical efficacy (46). Second-generation CARs incorporate a costimulatory signal, such as those from the CD28 and TNFR2 superfamilies, particularly CD28 and 4-1BB (TNFR2 superfamily), to increase the expansion, persistence, and activation of CAR-T cells (47). Third-generation CARs involve two distinct costimulatory molecules that work in concert with CD3ζ (48). Fourth-generation CARs introduce an inducible system in which T cells secrete specific cytokines to augment their functionality (49).

### 3.1 Different types of recognition chimeric domains

In combination with CAR-based tumor immunotherapy, antibody fragments or natural ligand pairs can also be harnessed to target CAR specificity, particularly in the context of treatments for autoimmune diseases (44, 50–53).

#### 3.1.1 Antibody-based CARs

**Figure 2:**
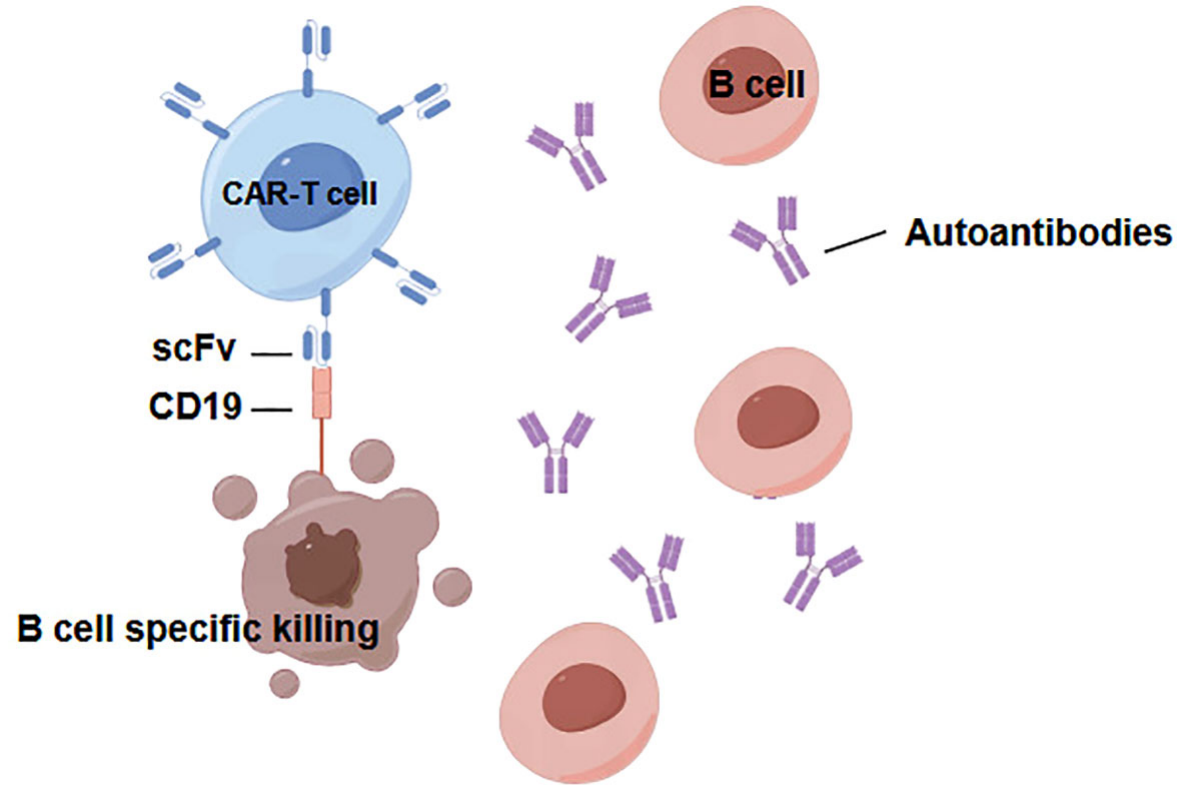
CD19 CAR-T cells are able to eliminate pathologic B cells in B cell-mediated diseases.

CD19 stands out as one of the most widely expressed proteins within the B lymphocyte lineage; it is induced during the differentiation of hematopoietic stem cells and subsequently downregulated upon the terminal differentiation of plasma cells (54). The extraordinary achievements of CD19-targeting CAR T-cells in maintaining long-term remission in B cell malignancies strongly demonstrate the enormous potential of CD19 CAR T-cell therapy in addressing a range of B- cell-mediated diseases (**Figure 2**). This promising avenue of therapy signifies a paradigm shift that has significant implications for managing B-cell -related conditions (55). In situations involving autoimmune disorders, including kidney diseases, the specific autoantibody target is often unknown. Owing to the expression of CD19 exclusively on B lymphocytes, cytotoxic CAR cells equipped with ScFvs targeting CD19 present compelling prospects for inhibiting the progression of immune-mediated glomerular diseases. However, a primary drawback of CD19 or CD20 CAR-based therapies is the broad depletion of B-cells, leading to a subsequent deficit in immunoglobulins, limiting the suitability of cellular strategies aimed at highly specific antibody-antigen recognition. In addition to the use of specific or nonspecific antibody-based domains for steering cytotoxic CAR cells toward pathogenic cells, ScFvs targeting autoantigens can also be used in CAR-based therapies for autoimmune renal diseases (56). These autoantigen-based ScFvs can guide CAR-Tregs to the inflammatory microenvironment, promoting their activation, proliferation, and subsequent expression of suppressive functions (56, 57).

#### 3.1.2 Natural recognition pair-based recognition designs in CAR therapies

**Figure 3:**
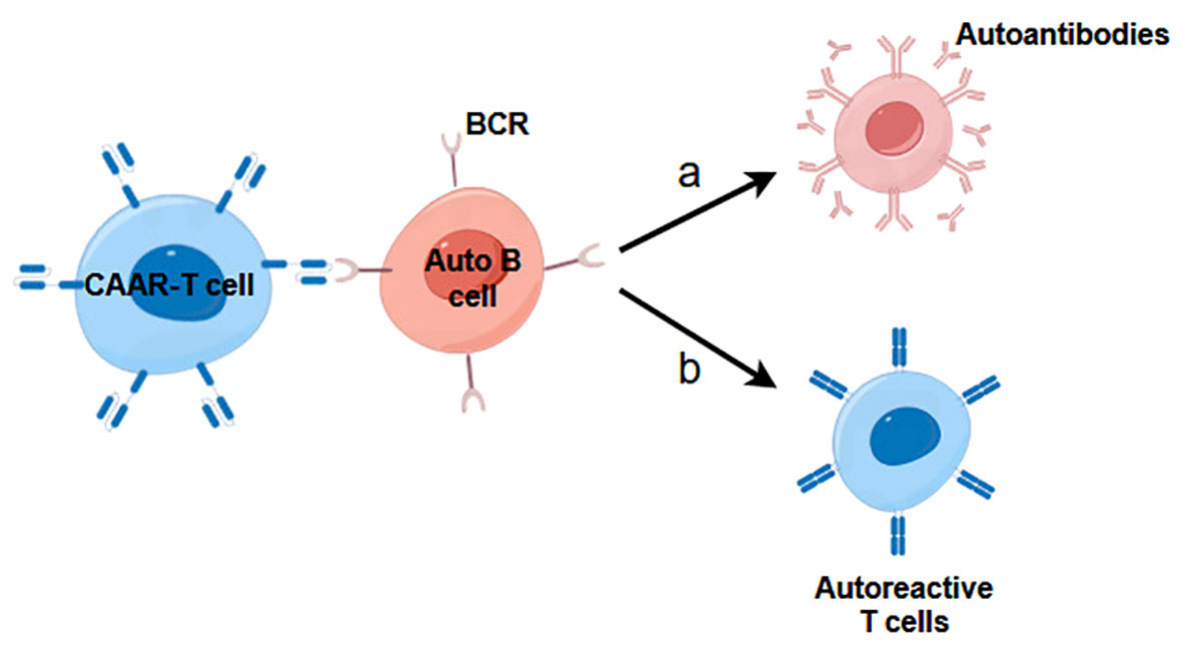
The mechanism of action of CAAR-T cells in immune-mediated kidney diseases. **(A)** CAAR-T cells inhibit B cells development and prevent them from secreting autoreactive antibodies. **(B)** CAAR-T cells prevent B cells from presenting autoantigens to autoreactive T cells, which leads to suppression of T cell-mediated kidney diseases.

In addition to the aforementioned antibody-based recognition strategies, an array of alternative natural recognition pairs can be harnessed in the design of CARs to address autoimmune diseases associated with TCRs or B cell receptors (BCRs). For example, the development of chimeric receptor modules utilizing the ectodomains of pMHC II complexes presents a potential avenue, allowing interactions with clonotypic TCRs of pMHC II-specific T cells (58). Moreover, the innovative concept of CAARs, which incorporate specifically engineered epitopes derived from autoantigens, represents a cutting-edge therapeutic modality for combatting autoimmune diseases by selectively targeting autoreactive B cells through the binding of autoantigens and BCRs (**Figure 3**) (59–61). This approach is designed to directly eliminate surface immunoglobulin memory B cells and indirectly eliminate short-lived plasma cells that produce disease-causing autoantibodies. Nevertheless, studies have demonstrated that CAAR cells can endure in the presence of soluble autoantibodies *in vivo*, suggesting that circulating autoantibodies do not result in Fc-mediated clearance of CAAR cells (62). The successful implementation of the CAAR/BAR strategy would suggest that this type of cell therapy may apply to immune-mediated kidney disorders where the autoantibody target is known.

## 4 CAR-based therapy in kidney diseases

In immune-mediated renal diseases, the traditional therapeutic approach, which typically involves steroids and other immunosuppressants, is gradually being transformed into more selective B-cell therapies. This shift has been propelled, in part, by the clinical success observed with B-cell depletion therapies (63). Given the substantial evidence from diverse preclinical models and the promising initial clinical results in systemic lupus erythematosus (SLE), there is a compelling rationale to further investigate the potential of CAR T-cell therapy in additional immune glomerular diseases (12, 51, 64). Diseases currently treated with a B-cell-depleting approach, such as membranoproliferative GN (MPGN), membranous nephropathy (MN), and ANCA associated vasculitis, could be strong candidates for CAR T-cell therapy (63). Given the comprehensive clearance of B cells, CD19 CAR T cells have demonstrated effectiveness in cancer contexts resistant to monoclonal antibodies, suggesting their potential efficacy in treating B-cell -mediated glomerular diseases even if monoclonal antibodies have shown minimal impact. Immune-mediated kidney diseases appear to be a promising area for exploring the potential of CAR T-cell therapy beyond oncology (**Table 1**).

**Table 1:** CAR-T therapies in preclinical and clinical studies of immune-mediated renal diseases.

Disease	Target antigen	Outcome	Reference
Lupus nephritis	CD19	Depletion of CD19+B cells, increased life span and alleviated diseases.	Kansal, R., et al. (64)
	CD19	Increased life span, effectively depletion B cells but without significant biochemical tests.	Jin, X., et al. (51)
	CD19	Effectively improved serious SLE disease manifestations within weeks, and presented the long-awaited cure.	Mougiakakos, D., et al. (65)
	CD19	Showed a significant decrease in the SLEDAI-2K score, proteinuria, anti-dsDNA levels at the end of 3 months after anti-CD19 CAR T therapy.	Mackensen, A., et al. (12)
Multiple myeloma-related renal impairment	BCMA/CD19	Highlighted the significant improvement in impaired renal function with CAR T-cell treatment for R/R MM.	Li, H., et al. (66)
	BCMA	Patients with impaired renal function exhibited a significant increase in eGFR from baseline.	He, S.L., et al. (67)
	BCMA	Renal function improved in certain patients following CAR-T treatment.	Sidana, S., et al. (68)
Light-chain amyloidosis	BCMA	Patient sustained hematological complete remission and attained a renal response.	Oliver-Caldes, A., et al. (69)
	CD19	Patient showed a profound hematological response six months after the therapy.	Korell, F., et al. (70)
	BCMA	Showed the viability of BCMA-CART cells therapy for patients with advanced AL amyloidosis.	Kfir-Erenfeld, S., et al. (71)
	SLAMF7	This mouse trial demonstrated the efficacy of SLAMF7 CAR-T cells in mediating potent anti-tumor effects.	Rosenzweig, M., et al. (72)

However, CAR T-cell therapy has adverse effects, such as cytokine release syndrome (CRS), immune effector-associated neurotoxicity syndrome (ICANS), tumor lysis syndrome (TLS), and acute kidney injury (AKI). To mitigate the risks of CRS and ICANS, a strategy of fractionated dosing can be employed. Concurrently, the prophylactic use of medications such as tocilizumab and corticosteroids is also a common strategy. Hunter et al. (73) reported the successful treatment of dialysis-dependent patients with CAR T-cells for relapsed/refractory DLBCL, which makes it a strong consideration in treating renal disease patients. Severe capillary leakage from the CRS leads to hypotension and multiorgan failure, and may lead to AKI in this population (74). Additionally, other factors, such as lymphodepleting therapy (fludarabine), dehydration, and nephrotoxic agents, may potentiate prerenal or other renal injuries. The incidence rate of AKI after CART-cell therapy ranges from 5% to 30%, with 9.8 times greater odds of developing AKI in patients with grade 3 or 4 CRS (74, 75). Recently, Leon-Roman J et al. (76) investigated the incidence, risk factors, and long-term outcomes of AKI in patients who have undergone CAR-T cell therapy for hematological malignancies. The study suggested that AKI is a frequent but typically mild adverse event following CAR-T cell therapy, with most patients experiencing a rapid recovery of kidney function within one month post-treatment. Male gender, history of CKD, and the development of CRS and ICANS are identified as independent risk factors for AKI. They also found that patients with previous CKD had higher risk of AKI development. The majority of CAR T cell therapy-related AKI remains reversible by treating the underlying cause, adequate hydration, supportive care, and tocilizumab or siltuximab.

### 4.1 Lupus nephritis

LN poses a formidable obstacle to effective treatment, a challenge significantly compounded by its inherent heterogeneity, an aspect rendered more conspicuous in the modern era of bioinformatics (77). In 2011, SLE was graced with the approval of the first biologic: belimumab. This fully humanized anti- BAFF monoclonal antibody (mAb) operates by obstructing the binding of BAFF with its corresponding receptors on B cells. Diseases such as neuromyelitis optica (NMO) are mediated by autoantibodies that target surface antigens. In contrast, SLE is characterized by class-switched antibodies against intracellular antigens, particularly nuclear components (78). The pathogenic potential of these antibodies lies in their ability to form immune complexes, ultimately leading to damage in various organs and tissues. However, current treatment approaches have limitations, as severe forms of SLE often exhibit resistance to treatment, and achieving long-term drug-free remission remains unrealized.

Researchers have developed CD19 CAR-T cells to eradicate aberrant B cells in two lupus mouse models (64). CD19 CAR-T cells effectively target and eliminate CD19+ B cells, resulting in reduced secretion of autoantibodies. This increased the lifespan of the two mouse strains, with most CD19-d NZB/W mice living up to 18 months after treatment, compared with the untreated group, which had all been euthanized at that point. They also measured proteinuria in lupus mice and reported that 5 months after CAR T-cell administration, the treated mice presented a significant reduction in or even disappearance of proteinuria, achieving clinical remission of kidney disease compared with the control group. They also showed that CD19-directed CAR T cells remained viable and functional several months after injection along with the effective elimination of autoantibody production. The examination of mouse serology subsequent to CAR T-cell treatment revealed a reduction in total IgM and IgG antibodies and decreased levels of anti-DNA IgG and IgM. Pathology of NZB/W renal tissue confirmed that the administration of CAR T cells diminished the size and degree of cellular infiltration in the glomeruli and largely prevented IgG deposits. The significant differences in pathology scores between CAR T cell–treated and control mice confirmed that anti-CD19 CAR T cells ameliorate a range of lupus symptoms. Furthermore, RNA analysis revealed that the modified T cells did not eradicate healthy B-cell populations, including splenic B-cell populations and bone marrow-resident plasma cells. These significant findings strongly suggest the potential of CD19 CAR-T cells to maintain functionality over extended periods and selectively eliminate transferred autologous CD19+ B cells without detrimental effects on other B cell populations within the bone marrow of murine lupus. Jin et al. (50) conducted an investigation involving the administration of the same anti-mouse CD19 CAR-T cells to MRL-lpr mice and explored various treatment strategies, including CAR-T and monoclonal antibody therapies, as well as second-generation CAR-T cells with different costimulatory motifs. Their research revealed enduring depletion of B cells in MRL-lpr mice following the administration of anti-CD19 CAR-T cells, with CAR-T cells featuring 4-1BB displaying enhanced therapeutic effects. Furthermore, they reported that pretreatment of mice with low-dose total body irradiation (TBI) significantly increased survival rates. Kidney histology also revealed less lymphocyte infiltration and less crescent deposition after the adoptive transfer of 4-1BB CAR-T cells, and granular immune complexes were reduced or absent.

These promising outcomes in mouse models provide a strong impetus for the clinical evaluation of CAR T cells for treating lupus diseases. These predictions were substantiated in the pioneering case of a patient with lupus, where the administration of anti-CD19 CAR T cells led to a notable resolution of the previously incurable disease (65). This study demonstrated that cellular engineering effectively improved severe SLE disease manifestations within several weeks, suggesting that CAR T-cell therapy might represent a long-awaited cure for SLE. In another study by Mackensen et al., five patients with SLEDAI-2K (with Systemic Lupus Erythematosus Disease Activity Index-2000) scores of 8–16 who had previously failed multiple therapies were enrolled; these patients presented significant decreases in the SLEDAI-2K score, proteinuria, and anti-dsDNA levels and increases in complement levels at 3 months after anti-CD19 CAR T-cell therapy. All the immunomodulatory agents were discontinued, resulting in drug-free remission in all five patients. Surprisingly, a posttreatment assessment revealed the reappearance of naive B cells a few months after CAR T-cell infusion, despite the absence of any recurrence of disease symptoms. The infusion of CAR T cells was effective, with only mild instances of CRS, thereby corroborating the notion that a reduced target cell burden could alleviate the severity of CRS. They also did not find a substantial decline in vaccine responses, indicating that CAR T-cell therapy primarily affects autoantibody-producing cells rather than all immunoglobulin producing cells. Extensive clinical trials and extended follow-up periods will be indispensable in determining whether the resolution of immunopathology is temporary or enduring. Owing to the limited number of patients treated, it is still premature to ascertain whether chronic B-cell aplasia may develop in some SLE patients (12).

Drawing from recent studies conducted in pemphigus vulgaris (PV), the enhancement of B-cell targeting by engineering CAAR-T cells could also be proposed for SLE. Nonetheless, the vast diversity of autoantibodies observed in SLE (approximately 180) implies the presence of numerous autoantigens that could be targeted (79). The development of CAAR-T cells aimed at these antigens could be compelling and valuable for evaluation in lupus mouse models, as they may present a promising approach for effectively depleting specific pathogenic B cells. The immune environment in SLE patients substantially diminishes the number and inhibitory function of Treg cells, thereby disrupting immune homeostasis (80). Therefore, replenishing the number of Treg cells and their related inhibitory functions through CAR-Tregs may effectively prevent SLE (81). Notably, Dall’Era et al. demonstrated that autologous Treg infusion increased Treg activation in inflamed skin and reduced infiltration by IFN-γ CD4+ effector cells in an SLE patient with skin involvement (82). Additionally, He J et al. conducted a study that revealed marked reductions in disease activity and increased numbers of Tregs in the blood of patients with SLE following treatment with low-dose interleukin-2 (83). These findings suggest that CAR-Treg therapy for SLE can restore immune tolerance and mitigate inflammation within affected tissues.

Several phase I clinical trials of CAR-T cells have been reported in refractory SLE patients (**Table 2**).

**Table 2:** The latest clinical trials of CAR-T for immune-mediated renal diseases.

Disease	Target antigen	NCT, status
SLE	CD19	NCT05988216, Recruiting NCT03030976,Phase I unknown
	BCMA/CD19	NCT06350110, Phase I,II not yet recruiting NCT05030779, Phase I unknown
Autoimmune kidney diseases (LN, AAV, MN, and IgG4-related diseases)	BCMA/CD19	NCT06285279, Phase I recruiting
ANCA associated vasculitis	BCMA	NCT06277427, Recruiting
	CD19/CD20	NCT06462144, Phase I not yet recruiting
Light-chain amyloidosis	BCMA	NCT06097832, Phase I not yet recruiting
	BCMA/CD19	NCT05978661, Phase I recruiting

### 4.2 Membranous nephropathy

MN is a significant cause of nephrotic syndrome (84). In 70% of patients, no underlying cause is identified, and the term primary (idiopathic) MN is used (85). The identification of the M-type phospholipase A2 receptor (PLA2R) as the primary target in MN in 2009 significantly advanced basic and clinical research in this field (1). MN is now recognized as a renal-limited autoimmune disease, with antibodies against PLA2R (aPLA2Rab) detected in 70% to 80% of cases and antibodies against thrombospondin type-1 domain-containing 7A (THSD7A) in 2% to 5% of patients (86) ^(^ 87 ^),^. The clinical outcomes of patients afflicted with MN differ significantly. Approximately one-third of patients experience spontaneous remission within a year after diagnosis, while an additional 20–30% progress to renal failure within a decade (88). Despite these advancements, the management of MN remains a contentious subject. Historically, cyclophosphamide-based regimens have been the standard of care, demonstrating efficacy in preventing advanced kidney failure, albeit at the cost of increased risk of malignancy for patients (89). Treatment with calcineurin inhibitors (CNIs) such as cyclosporine or tacrolimus can induce high rates of remission but is associated with an elevated risk of relapse and concerns surrounding renal toxic effects during prolonged use (90). Therapeutic regimens involving CD20-targeted agents, such as rituximab, are generally well tolerated. However, only 60%-70% of patients achieve sustained clinical remission, and their effectiveness in preventing kidney disease progression has not been fully demonstrated (90). Moreover, rituximab treatment can potentially lead to resistance toward apoptosis, ADCC, and the downregulation or loss of CD20 (91), thereby reducing treatment efficacy. Furthermore, it may impact preexisting protective antibodies and undermine the body’s capacity to develop an immune response against external organisms.

Advances in understanding specific antigens implicated in MN pave the way for antigen-specific treatment strategies that target the immune system’s disease mechanisms while preserving protective immunity. A promising therapeutic approach to eradicate pathogenic autoreactive B cells involves the development of CAAR-T cells, a modification of CAR-T cells (92). This novel therapy offers the advantage of generating long-term memory CAR-T cells, ensuring sustained efficacy against newly produced target cells and eliminating the need for repetitive dosing (93). The efficacy of this method was initially evaluated in an animal model of PV, where T cells expressing a CAAR comprising the PV autoantigen desmoglein 3 fused to CD137-CD3ζ effectively eliminated autoreactive B cells specific to desmoglein 3 (62). Furthermore, CAAR T cells effectively targeted their designated antigens even in the presence of circulating anti-desmoglein 3 antibodies, without manifesting any notable off-target effects. In a subsequent study, researchers conducted preclinical evaluations of the pharmacodynamics and toxicity of this CAAR. They reported that desmoglein 3-CAAR T cells selectively depleted primary human desmoglein 3-specific B cells from PV patients, yielding favorable outcomes in a live animal model of PV (59).

The use of CAAR T cells in the treatment of MN represents an innovative approach. The binding sites of antibodies in the antigens PLA2R1 and THSD7A have been extensively identified, including the level of individual antigen domains and smaller epitopes. Consequently, to achieve the ideal intermembrane distance at the immunological synapse, it appears reasonable to merge smaller fragments of the target antigen to the chimeric receptor, a strategy that holds promise for effectively targeting and eliminating a considerable population of autoreactive B cells (93). However, circulating autoantibodies present a potential obstacle that must be overcome to ensure an effective CAAR T-cell response. When binding to the antigenic region of the CAAR, these autoantibodies have the potential to deactivate or even clear the administered CAAR T-cells. Addressing this challenge could involve the immunoadsorption of specific autoantibodies (92), creating a favorable time window for enhanced CAAR T-cell activity. In summary, CAAR T-cell therapy is a promising therapeutic approach for patients with MN.

### 4.3 ANCA-associated vasculitis

Microscopic polyangiitis (MPA) and granulomatous polyangiitis (GPA) are both types of ANCA-associated vasculitides (AAVs) that are characterized by autoimmune necrotizing vasculitides affecting small vessels such as capillaries, veinules, or arterioles. Importantly, kidney disease is a common manifestation of AAV and serves as a significant predictor of mortality. Individuals with glomerular filtration rates (GFRs) < 50 mL/min have a high risk of death or kidney failure within 5 years (94). This information underscores the critical impact of kidney involvement in AAV and the associated risk for adverse outcomes. The typical renal manifestation of AAV is rapidly progressive glomerulonephritis, characterized by a rapid decline in renal function over days to months. The pathogenicity of ANCAs has been indicated by numerous *in vitro* experiments (95), which have demonstrated their ability to bind to their target antigens and induce neutrophil activation. This activation ensues after neutrophils are primed by proinflammatory cytokines, including TNF-α, IL-8, and TGF-β. Furthermore, additional data have indicated that ANCAs can trigger the formation of neutrophil extracellular traps (NETs), which in turn activate dendritic cells and autoreactive B-cells. These activated cells can then present cellular autoantigens such as PR3 or MPO, contributing to the autoimmune response (96).

Abundant evidence indicates extensive engagement of B cells in the pathogenesis of AAV. This includes B-cell activating factor release by ANCA-activated neutrophils, epitope spreading in ANCA production leading to the generation of pathogenic antibodies, the overexpression of ANCA autoantigen genes, and subsequent cascades culminating in the production of pathogenic ANCAs by B cells and plasma cells. These findings collectively emphasize the significant role of B cells in the development and progression of ANCA-associated vasculitis (97). The landmark studies on rituximab in AAV (98) (99), and the comparison of rituximab versus cyclophosphamide in AAV (100) have underscored the importance of B cells in the management of PR3- and MPO-ANCA vasculitis. Rituximab, a monoclonal antibody directed against CD20-B cells, has become a cornerstone in treating these conditions, highlighting the importance of targeting B cells in AAV. However, it is important to note that rituximab follows an exponential decay trend in its pharmacokinetics and typically needs repeated administration to maintain therapeutic levels. The use of anti-CD20 monoclonal antibodies has demonstrated suboptimal efficacy, possibly due to the transient and incomplete depletion of B cells. Notably, CD20 is not expressed in plasma cells, and antibody therapy primarily targets circulating B cells. In contrast, self-reactive B cells may persist in the lymph nodes, renal tubules, and bone marrow (101). Therefore, CAR-T cells can potentially deplete all target cells effectively in circulation and tissues. This feature of CAR-T cell therapy presents a possibility for the comprehensive elimination of self-reactive B cells in AAVs (102).

CAR T cells hold promise as a potential treatment approach for AAV, especially considering the dysregulation of B-cell homeostasis in this disease and the favorable outcomes demonstrated with rituximab. By targeting the B cells responsible for the production of pathogenic autoantibodies involved in the pathogenesis of AAV, such as ANCAs, it may be possible to engineer CAAR T cells that specifically eliminate these pathogenic cells while preserving protective B cells. This targeted approach has the potential to offer a highly tailored and effective treatment strategy for AAV, addressing the specific cellular mechanisms driving the disease. Recently, Dörte Lodka et al. (103) investigated the potential of CD19 CAR T cells in treating AAV via a preclinical mouse model of MPO-AAV. CD19 CAR T cells effectively migrated to and persisted in the bone marrow, spleen, peripheral blood, and kidneys for up to 8 weeks. These cells deplete B cells and plasma blasts, enhance the decrease in MPO-ANCA, and most importantly, protect mice from necrotizing crescentic glomerulonephritis. In contrast, control CAR T cells did not have the same protective effects. These findings may encourage further exploration of CAR T cells as a treatment for ANCA vasculitis patients, to achieve drug-free remission. Furthermore, some studies have revealed defective Treg function in patients with AAV, manifested by a diminished or absent inhibitory effect on T-cell proliferation (104–106). The molecular mechanism underlying this phenomenon seems to be linked to the upregulation of miR-142-3p in memory Tregs (CD4+CD45RO+CD25+CD127-), leading to the inhibition of cAMP synthesis via the targeting of ADCY9 (107). Clinical research on kidney transplantation and experimental investigations involving other immune-mediated renal disorders have focused primarily on polyclonal Treg cells. There is potential for the design of CAR-Tregs targeted to specific antigens and tissues, which could mitigate inflammation and promote physiological tissue repair. This targeted approach offers a promising avenue for immune modulation in AAV and related autoimmune conditions, addressing the underlying immunological dysregulation.

### 4.4 Monoclonal immunoglobulin-mediated renal disease

#### 4.4.1 Multiple myeloma-related renal impairment

Multiple myeloma (MM) is a malignant plasma cell disorder that can damage multiple organs, leading to bone destruction, anemia, hypocalcemia, and renal impairment (RI). The International Myeloma Working Group (IMWG) defines the RI in MM as either a serum creatinine level exceeding 2 mg/dL (170 μmol/L) or a reduced creatinine clearance (CrCl < 40 mL/min) (108). RI is present in up to 50% of patients with MM at the time of diagnosis (108–110), and approximately 2%–4% of these patients will require dialysis (111). Research has demonstrated that the RI is related to decreased overall survival and an elevated risk of early mortality in MM patients (112–114). Some studies have suggested a correlation between outcomes and the extent of decline in the estimated glomerular filtration rate (eGFR) (111, 113).

Renal injury in individuals with MM can be predominantly attributed to the harmful impact of monoclonal free light chains (FLCs) on the glomeruli and renal tubules (108, 115). Excessive production of monoclonal FLCs in MM can exceed the absorptive and metabolic ability of proximal renal tubule cells (115). The persistence of FLCs in the proximal tubules may trigger apoptotic molecular pathways, resulting in inflammation and fibrosis. Furthermore, when unabsorbed FLCs reach the distal nephron, they can interact with the Tamm-Horsfall protein, forming precipitates and casts, subsequently causing tubular blockage and an inflammatory response (115). These findings represent the primary pathophysiological mechanisms underlying the development of light-chain cast nephropathy. Additionally, other pathologies mediated by monoclonal FLCs may co-occur with proximal tubule fibrosis and monoclonal cast nephropathy, such as light-chain amyloidosis, monoclonal immunoglobulin deposition disease, and acute tubular necrosis (110, 111).

Immunotherapy employing CAR T cells designed to target BCMA on myeloma cells has garnered regulatory approval for treating relapsed or refractory MM and has significantly improved the prognosis of patients who have undergone at least four prior lines of therapy and are considered triple-class refractory. In a small study involving seven patients with relapsed or refractory MM and an eGFR of 15–29 mL/min per 1.73 m², CAR T-cell therapies directed at either BCMA alone or a combination of BCMA and CD19 demonstrated a 100% overall response rate and 100% renal response rates. The median time to the first renal response was 9 days, and the median time to the best renal response was 32 days after CAR T-cell therapy. These findings highlight the significant improvement in impaired renal function with effective CAR T-cell therapy for R/R MM (66). An additional retrospective report analyzed 59 patients from two clinical trials, categorizing them into two cohorts based on renal function, with a cutoff eGFR of 90 ml/min/1.73 m² (67).In patients with impaired renal function, the eGFR at the 6th month post CAR T-cell infusion significantly increased from baseline, particularly in those with light chain-related conditions. Notably, response rates were comparable between the two cohorts; however, patients with normal renal function had longer progression-free and overall survival. Furthermore, a recent retrospective multicenter observational study, which included patients with and without RIs who underwent treatment with BCMA CAR-T cells at 11 medical centers in the U.S., assessed the real-world outcomes of RRMM patients with RIs. The study cohort comprised 214 patients, with 28 (13%) presenting RIs, and among them, 11 patients had severe RIs (CrCl of <30 ml/min or requiring dialysis). Renal function improved in certain patients following CAR-T therapy; notably, no renal function deterioration was observed in any patients with preexisting RIs. The response rates (93% vs. 82%) and survival outcomes (median progression-free survival: 9 vs. 8 months, p=0.26) were not significantly different between patients with and without RIs (68).Achieving durable cures through anti-BCMA CAR-T cell therapy in myeloma is uncommon. A possible explanation is that a minor subset of minimal residual myeloma cells relapse. After treatment with BCMA-CAR-T cells, the remaining myeloma cells presented fewer differentiated features and expressed stem-like genes, including CD24. Therefore, CAR-T cells targeting CD24 are designed to recognize minimal residual MM cells and block the CD24-Siglec-10 pathway inducing tumor cell clearance by phagocytic macrophages. This intervention also activates inflammatory and antitumor signaling, thus promoting more durable remission in MM patients (116). In conclusion, CAR-T therapy is viable for patients with RIs, who exhibit a safety and efficacy profile comparable to those without RIs.

#### 4.4.2 Light-chain amyloidosis

Primary light chain amyloidosis (AL) is a rare form of monoclonal plasma cell (PC) disorder, distinguished by the systemic accumulation of misfolded immunoglobulin light chain (LC) protein products in various organs, leading to the formation of insoluble fibrils. These fibrils are produced by clonal malignant PCs, which may occasionally experience overproliferation (117–119). While AL is closely related to MM, the plasma cell burden is typically small, with a median of 5–10% clonal plasma cells. The accurate diagnosis of this condition requires the histologic confirmation of amyloid deposition and typing, combined with the presence of monoclonal plasma cell disorders via the detection of a monoclonal protein in the serum, urine, or bone marrow (120). The kidney and heart are the most frequently affected organs, although the liver, spleen, soft tissue, peripheral and autonomic nerves, and gastrointestinal tract may also be affected, albeit less commonly (121). Most individuals with AL amyloidosis (approximately 75%) present with proteinuria, and among them, half present with nephrotic syndrome at the time of diagnosis. Nearly half of the patients had elevated serum creatinine levels, and only 20% had levels that surpassed 2.0 mg/dL. The mortality rate is primarily linked to the extent of cardiac involvement, whereas kidney survival is determined by the degree of kidney involvement (122).

The primary goal of therapy is to eliminate the amyloidogenic plasma cell clone, aiming to achieve a deep hematological and organ response crucial for improving overall survival (OS) (123, 124). In recent years, effective drugs used to treat multiple myeloma, such as bortezomib and lenalidomide, have been approved for AL amyloidosis therapy (125). The ANDROMEDA trial demonstrated that adding daratumumab led to better hematologic and target organ (cardiac and renal) responses than bortezomib-based treatment alone (126). The combination of daratumumab with cyclophosphamide, bortezomib, and dexamethasone (Dara-CyB or D) has been identified as a promising first-line treatment for newly-diagnosed AL amyloidosis (127). However, inducing minimal residual disease negativity in advanced or refractory disease is challenging and rare. There is an urgent need for alternative effective therapies for these patients.

CAR-T cells have changed the treatment landscape for B-cell neoplasms, providing efficacy in both frontline and relapsed/refractory settings, and are an evolving strategy for the treatment of MM. Research has revealed high expression of BCMA on plasma cells associated with AL amyloidosis (72), with a study of 34 bone marrow biopsies from affected patients demonstrating a median BCMA expression of 80% (128). Therefore, CAR-T cells targeting BCMA show promise in treating AL amyloidosis patients. A case report highlighted the use of BCMA CAR-T therapy in a patient with AL amyloidosis and renal involvement. The patient experienced significant symptomatic relief, accompanied by a profound hematological response and negativity for residual disease according to bone marrow flow cytometry after 3 months of treatment (69). After 1 year, the patient experienced complete hematological remission and achieved a renal response, as evidenced by a remarkable 70% reduction in proteinuria. Another patient, a 71-year-old male diagnosed with systemic AL amyloidosis and marginal zone lymphoma, received third-generation CAR-T cell therapy targeting CD19. The use of CAR-T-cell therapy was well tolerated, even in the presence of heart and kidney amyloid involvement, with only early low-grade procedure-specific toxicities noted. Following treatment, the patient showed a progressive decrease in IgM, kappa light chain, and the kappa-to-lambda light chain ratio, resulting in a profound hematological response six months after therapy (70). Based on the seminal results of the scholarly work involving BCMA-CAR T (129), a study reported ex vivo data utilizing their scholarly conceived BCMA-CAR T, denoted HBI0101, and effectively administered it to patients afflicted with RR AL with multiple organ involvement. This study shows the feasibility of BCMA-CAR T-cell therapy for patients with advanced AL amyloidosis (71). As the clinical trial continues, future CAR T-cell clinical trials and real-world empirical evidence involving patients with AL amyloidosis are anticipated, contributing to a more robust demonstration of the safety and effectiveness of utilizing BCMA-CART for the improvement of AL amyloidosis.

The consistent expression of SLAMF7 on AL amyloidosis plasma cells has prompted notable advancements in the field. The successful injection of SLAMF7 CAR T cells into mouse models with SLAMF7-expressing AL amyloidosis resulted in a remarkable reduction in tumor size compared with that of control T cells. This compelling *in vivo* trial demonstrated the efficacy of SLAMF7 CAR-T cells in mediating potent antitumor effects in the context of AL amyloidosis (72). Currently, two phase 1 clinical trials evaluating the effectiveness of SLAMF7 CAR-T-cell products in MM are underway (ClinicalTrials.gov Identifiers: NCT04142619 and NCT03710421). These developments indicate the potential for exploring clinical trials involving SLAMF7 CAR-T cells for treating AL amyloidosis, indicating a promising avenue for further therapeutic research and practice.

## 5 Questions about the application of CAR-based therapies in kidney diseases

The utilization of genetically modified T cell has emerged as a promising therapeutic avenue for treating immune-mediated kidney diseases, and has the potential to effectively eliminate autoreactive cells without inducing significant off-target toxicity (62). While preclinical studies in autoimmune diseases have achieved favorable outcomes, the application of CAR-based therapy in the clinical management of kidney diseases faces numerous critical challenges. These include the stability, durability, safety, effectiveness, and scalability of the therapy, all of which must be overcome before CAR-based therapy can be confidently translated into clinical application for kidney diseases.

CRS represents a significant and challenging type of clinical toxicity associated with CAR T-cell therapy. Initial clinical evidence from CAR T-cell therapy in SLE patients indicates that the manifestation of CRS may be less severe in noncancer contexts, particularly where the target cell burden is comparatively low. However, the extent of this milder CRS in larger SLE trials and in the context of other autoimmune kidney diseases warrants comprehensive assessment. Notably, regarding this concern, Tregs have emerged as a potentially safer option than cytotoxic T cells (130). The incorporation of suicide genes to induce CAR T-cell death is one of the pioneering clinical applications of gene transfer technology in human subjects. Several suicide switches have been elucidated, and efforts are underway to envision more promising switches for the future. For example, potential developments may involve switches that independently trigger cell death if FoxP3 expression is diminished in CAR-Tregs or if the expression of IL-17 and/or other proinflammatory cytokines is increased (131, 132). Moreover, the risk of CAR-Treg-mediated bystander suppression of antitumor or anti-infectious effects is an important consideration that needs to be studied in preclinical models (133).

Whether lymphocyte depletion is necessary before CAR-T cell therapy for kidney disease remains an unresolved issue, particularly considering the lower target cell burden than that of malignant tumors. A definitive determination regarding lymphocyte depletion could promote patient acceptance of this innovative therapy, especially at earlier stages of the disease (134). Additionally, considering that the primary goals of CAR-based therapy are low cost and high efficiency, developing the optimal dosage strategy is a key issue. Compared with those for hematological malignancies, CAR T-based therapies for autoimmune kidney diseases remain relatively unexplored, with few studies in this area.

The challenge of CAR T -cell exhaustion arises from prolonged exposure to specific antigens and the confluence of an immunosuppressive microenvironment, presenting a significant obstacle to sustaining effector function and clinical efficacy. Overcoming this hurdle remains imperative in optimizing CAR T-cell therapy. A potential strategy to address this lies in targeting intrinsic T-cell pathways, with diverse methods available to modulate their activity. These include blocking exhaustion-inducing signaling, quelling downstream effectors, reversing inhibitory signals into stimulatory signals, and refining the design of the CAR itself. Encouragingly, these innovative approaches have displayed considerable promise, and as they have progressed through clinical trials, their safety and efficacy are eagerly awaited (135).

The occurrence of AKI in approximately 20% of patients receiving CAR-T cell therapy in clinical trials has drawn widespread attention. Various pathophysiological mechanisms, including CRS, hemophagocytic lymphohistiocytosis (HLH), tumor lysis syndrome, serum cytokines, and inflammatory biomarkers, intervene in this process. However, with prompt identification and adept management of these CAR-T cell-related complications, the prevalence and severity of AKI are mitigated, with most patients exhibiting recovery of kidney function within 30 days (136). The challenge of AKI following CAR-T cell therapy will undoubtedly become a perpetual task for nephrologists, underscoring the need for ongoing research and clinical intervention in this domain.

Although CAR T-cell therapy has shown remarkable success in treating certain types of cancers, there have been reports of patients who developed T-cell lymphomas after receiving this treatment. The mechanism underlying the development of T-cell lymphoma in patients treated with CAR T-cell therapy is not fully understood and is a subject of ongoing research. T-cell lymphomas may be induced under unusual settings, such as retroviral activation of JAK kinase (137) and at very high insertion copy numbers via a transposon system for CAR gene delivery (138). The FDA has issued safety alerts regarding the risk of T-cell malignancy following treatment with BCMA-directed or CD19-directed autologous CAR T-cell therapies. This has led to a heightened focus on the long-term safety and monitoring of patients who have received CAR T-cell therapy. Importantly, that while the development of T-cell lymphoma is a serious concern, it appears to be a rare event based on current data. The benefits of CAR-T cell therapy for certain diseases still outweigh the risks for many patients, particularly those with advanced diseases who have not responded to other treatments (139). Gurney M et al. reported that age and thrombocytopenia, recognized as intuitive baseline indicators, can effectively stratify the risk of myeloid neoplasms in patients who undergo CAR T-cell therapy. These factors hold significance regardless of clonal hematopoiesis, aiding the counseling process and guiding surveillance strategies. Although higher than posttransplant rates for MM and lymphoproliferative disorders, post CAR T-cell neoplasm events could not be directly attributed to CAR T-cell exposure because all patients received prior cytotoxic therapies (140).

## 6 Conclusion

With the emergence of CAR-T cell therapy, the landscape of treating immune-mediated kidney diseases has been irrevocably transformed. The use of CAR-T cells as a promising alternative to traditional treatments is predicated on their remarkable capacity for highly efficient and robust target cell depletion, coupled with their comparatively lower incidence of side effects than conventional drugs. Nevertheless, exploring the full potential of CAR-based cell therapies requires comprehensive preclinical and clinical studies, alongside addressing the difficult challenges of designing target recognition systems. The essential problem revolves around refining the recognition domains to encompass various autoimmune-specific targets, a critical attempt to optimize the efficacy of CAR-based treatments. Notably, the lack of studies focused on CAR T-cell therapy for immune-mediated kidney diseases underscores the pioneering nature of the research mentioned above, laying the foundation for the realization that a novel therapeutic strategy is on the brink of clinical fruition.
